# Effectiveness of personalized meal recommendation in improving dietary behaviors of Chinese community-dwelling elders: study protocol for a cluster randomized controlled trial

**DOI:** 10.1186/s13063-023-07865-1

**Published:** 2024-04-11

**Authors:** Zidu Xu, Xiaowei Xu, Lianglong Sun, Zhen Guo, Jianqiang Lai, Lin Kang, Jiao Li

**Affiliations:** 1 School of Nursing, Columbia University, New York, NY USA; 2 Institute of Medical Information, Chinese Academy of Medical Sciences and Peking Union Medical College, 3rd Yabao Road, Beijing, 100020 Chaoyang District China; 3 Chinese Center for Disease Control and Prevention, National Institute for Nutrition and Health, Beijing, China; 4 Department of Geriatrics, Peking Union Medical College Hospital, Beijing, China

**Keywords:** Elderly nutrition, Personalized meal recommendation, Community, Study protocol, Randomized controlled trial

## Abstract

**Background:**

Inappropriate eating behaviors, particularly a lack of food diversity and poor diet quality, have a significant impact on the prognosis of certain chronic conditions and exacerbate these conditions in the community-dwelling elderly population. Current dietary interventions for the elderly have not adequately considered the nutritional needs associated with multiple chronic conditions and personal dietary preferences of elderly individuals. A personalized recommendation system has been recognized as a promising approach to address this gap. However, its effectiveness as a component of an elderly-targeted dietary intervention in real-world settings remains unknown. Additionally, it is unclear whether this intervention approach will be user-friendly for the elderly. Therefore, this study aims to examine the effectiveness of a personalized meal recommendation system designed to improve dietary behavior in community-dwelling elders. The implementation process in terms of System usability and satisfaction will also be assessed.

**Methods:**

The trial has been designed as a 6-month, non-blinded, parallel two-arm trial. One hundred fifty community-dwelling elders who meet the eligibility criteria will be enrolled. Subjects will be allocated to either the intervention group, receiving personalized meal recommendations and access to corresponding food provided as one component of the intervention, as well as health education on elder nutrition topics, or the control group, which will receive nutritional health education lectures. Outcomes will be measured at three time points: baseline at 0 months, 3 months, and 6 months. The primary outcomes will include dietary diversity (DDS) and diet quality (CDGI-E) of enrolled community-dwelling elders, representing their dietary behavior improvement, along with dietary behavior adherence to recommended meals. Secondary outcomes will measure the perceived acceptability and usability of the personalized meal recommendation system for the intervention group. Exploratory outcomes will include changes in the nutritional status and anthropometric measurements of the community-dwelling elders.

**Discussion:**

This study aims to examine the effectiveness, acceptability, and usability of a personalized meal recommendation system as a data-driven dietary intervention to benefit community-dwelling elders. The successful implementation will inform the future development and integration of digital health strategies in daily nutrition support for the elderly.

**Trial registration:**

Chinese Clinical Trial Registry ChiCTR2300074912. Registered on August 20, 2023, https://www.chictr.org.cn/showproj.html?proj=127583

**Supplementary Information:**

The online version contains supplementary material available at 10.1186/s13063-023-07865-1.

## Administrative information

**Table:** 

Title {1}	Effectiveness of personalized meal recommendation in improving dietary behaviors of Chinese community-dwelling elders: study protocol for a randomized controlled trial
Trial registration {2a and 2b}	Chinese Clinical Trial Registry ChiCTR2300074912, registered on August 20, 2023.
Protocol version {3}	Issue Date: August 18, 2023 Protocol Version: 8^th^ version This manuscript details the protocol on the 8^th^ version approved on August 18 2023.
Funding {4}	This study was supported by Chinese Academy of Medical Sciences Innovation Fund for Medical Sciences (No. 2021-I2M-1–056) and Key R & D plan of Hunan Province (No. 2021SK2024)
Author details {5a}	Institute of Medical Information, Chinese Academy of Medical Sciences and Peking Union Medical College Columbia University, School of Nursing National Institute for Nutrition and Health, Chinese Center for Disease Control and Prevention Department of Geriatrics, Peking Union Medical College Hospital
Name and contact information for the trial sponsor {5b}	Trial Sponsor: Contact name: Jiao Li Email: li.jiao@imicams.ac.cn
Role of sponsor {5c}	The funding organization did not participate in the design of the study and was not involved in data collection, management, analysis, interpretation, manuscript preparation, or the decision to submit the findings.

## Introduction

### Background and rationale {6a}

China is stepping into a super-aging society with an elderly population over 60 years old expected to take the proportion of 28% by the year 2040 [1]. Keeping a healthy dietary behavior is a crucial step to promote healthy aging, as it plays a significant role in regulating metabolism, reducing age-related comorbidities and functional impairments, and facilitating adequate nutritional intake [2, 3]. However, with a higher chance of multiple chronic conditions (MCCs), functional disability, and limited activities of daily living (ADLs) capacity, the elderly are less likely to keep healthy eating behaviors. Specifically, faced with difficulties in food preparation, limited access to fresh and nutritious foods, and reduced appetite and chewing function, it is difficult for the elderly to consume a wide range of food groups to achieve high dietary diversity [3–5]. The diet quality of the elderly population is also a concern, which not only refers to the adequate achievements of essential nutrients and food variety but also incorporates avoiding unhealthy food choices to be a good alignment in dietary guideline recommendations [6, 7]. Low dietary diversity and diet quality can detrimentally affect the prognosis of certain MCCs and exacerbate the health conditions of the elderly population [5, 7, 8], thus leading to a self-perpetuating cycle of deteriorating health conditions and unhealthy diet. So far, inappropriate eating behaviors have put over 10% Chinese elderly population at risk of malnutrition [9].

Currently, 80% of the Chinese elder population (> 65 years) are now community-dwellers (i.e., living outside nursing homes or hospitals) [10], and dietary interventions targeting the elderly population are encouraged to be placed in community settings to enhance public health impact and resource accessibility. Existing community-based or home-based nutrition support services provide uniformed meal services or nutrition education to help the elderly population get access to nutritious food or increase produce intake [11, 12] yet are unable to meet the personal needs of elderly individuals, resulting in inadequate penetration and effectiveness [2, 12]. The eating behaviors of older adults are influenced by various factors, such as social and cultural contexts, pathophysiological conditions, and physiological changes. This indicates that dietary interventions targeting the elderly population should take a comprehensive consideration of specific nutritional requirements associated with MCCs, personal dietary preferences, medication usage, and their degrading functions of chewing and tasting. In addition, more than 50% of Chinese community-dwelling elders have limited nutrition literacy [13, 14], which hinders them to make informed food choices and achieve a well-rounded diet merely supported by meal services [15, 16].

A personalized food recommendation system aims to utilize comprehensive personal information and food knowledge to provide customized food recommendations [17, 18]. This system has been successful in improving the eating habits of hospitalized elderly patients and offering online restaurant recommendations to the general population [19, 20]. However, current food recommendation systems have yet well address the complex needs of elderly individuals in terms of nutrition, preferences, and ease of use [3, 21, 22]. These elements, however, are the key to community-based daily nutrition support targeted at the elderly population [2, 3]. To address this gap, we developed a personalized meal recommendation system to assist community-dwelling elders in dietary behavior improvement. This system is driven by the knowledge learned from the survey data of community-dwelling elders’ health status and history of diseases and personal dietary preferences, added with information regarding food composition and elderly nutrition guidelines [23]. The simulation experiments results provide evidence that this system is promising in improving the diet quality and food diversity of community-dwelling elders compared to their history of eating behaviors [23]. However, no practical interventions have been developed based on this system to incorporate all essential considerations mentioned above to improve the eating behaviors of community-dwelling elders. Therefore, it is still unclear about the clinical or public health impact of this innovative dietary intervention on the elders’ dietary behaviors in real-world community settings. The acceptability and user experience of implementing this intervention also remains to be articulated.

### Objectives {7}

To address the existing challenges in managing dietary behaviors among the Chinese community-dwelling elders, we have developed a personalized dietary behavior intervention pipeline featuring the automated generation of meal recommendations. The objectives of this trial are (1) to evaluate the effectiveness of personalized meal recommendations on primary outcomes of dietary behavior changes in community-dwelling elders during a 6-month intervention period, while also examining its impact on the nutritional status and anthropometric measurements of the elderly as additional outcomes, and (2) to assess the implementation process of our personalized meal recommendation system as a dietary intervention, including its perceived acceptability and usability as secondary outcomes.

### Trial design {8}

This experimental study expected to run from July 2023 to September 2024 will follow a non-blinded, paralleled, two-arm pre-test post-test design including 150 community-dwelling elders. This trial will be conducted simultaneously in six community health service centers within Nanhu district, Jiaxing City, located in Zhejiang province, China. Following a cluster randomization design, intervention and control sites will be allocated in a 1:1 ratio based on a randomized allocation sequence generated by a single investigator using R 4.2.0 The intervention period is expected to last for 6 months, following a preparation and recruitment phase. Effect measurements will be conducted at three time points: at the beginning, after 3 months, and after 6 months of the intervention. Implementation measurements will be carried out throughout the study. The trial design follows the SPIRIT checklist (Additional file 1).

## Methods: participants, interventions, and outcomes

### Study setting {9}

The study settings will be established in six community health service centers, strategically located in distinct geographical areas within the Nanhu district. These centers were chosen for their accessibility and convenience for device deployment. The participants are expected to be residents of these respective communities. In this trial, the healthcare professional team consisted of informatics scientists from the Institute of Medical Information, nutrition scientists from Chinese Center for Disease Control and Prevention, and clinicians from Peking Union Medical College Hospital. Participant recruitment and outcome measurements will be performed in the community centers, where community health service centers and elderly-serving canteens are located.

### Eligibility criteria {10}

Individuals are eligible if they meet the following inclusion criteria: (1) aged over 65, (2) being able to communicate independently, (3) able to make independent food choices; (4) able to access community sites to receive interventions; (5) able to take meals independently. Participants would be excluded if they are (1) diagnosed with cognitive impairment, indicated by a Mini Mental State Examination (MMSE) score less than 24, or (2) having dietary restrictions strictly mandated by medical prescriptions due to advanced severity of chronic conditions. This includes but is not limited to conditions like end-stage renal disease requiring strict electrolyte management or conditions necessitating highly restrictive diets. Individuals with common chronic conditions such as diabetes, hypertension, and hyperlipidemia are eligible, as they are expected to benefit from the personalized meal recommendations. Our proposed interventions are tailored to support the dietary management of these conditions, aligning with standard dietary guidelines without imposing severe conflicts.

### Who will take informed consent? {26a}

Eligible elderly individuals will be invited to attend the enrollment session, where trained social workers will explain the study in detail. Considering the potential literacy and functional limitations in this demographic, these social workers will use simplified language and visual aids to facilitate comprehension. Participants will receive a consent package containing clear information about the study’s purpose, procedures, risks, and benefits, as well as the assurance of the confidentiality of their data, which will be accessible only to investigators. They will have ample time for review and understanding. Social workers will answer questions and address concerns, ensuring full understanding before participants sign the informed consent forms. This process will occur before any allocation or baseline assessment.

### Additional consent provisions for collection and use of participant data and biological specimens {26b}

The informed consent has included participants’ permission for the use of collected data. Further analysis of biological specimens does not apply to this study.

## Interventions

### Explanation for the choice of comparators {6b}

Comparators were dietary interventions in the form of elderly-targeted nutritional health education in the format of a series of lectures. This is because the effectiveness of nutritional health education has been proved in relevant studies focusing on elderly nutritional support [21]. In addition, the development of our meal recommendations in integrated intervention also refers to the nutrition guidelines that the lecture materials are derived from.

### Intervention description {11a}

#### Usual nutrition support for the control group

Participants in the control group will receive intervention in the format of nutrition and health education based on Chinese geriatric nutrition guidelines and consensus [13]. The learning objective of health education is to provide community-dwelling elders with essential knowledge about nutrition and geriatric health management. Simultaneously, it aims to stimulate their internal motivation to adopt and maintain healthy eating habits in the long term. Table 1 presents the main content of the health education, including (1) food and nutrients, (2) senior nutrition guidelines, and (3) nutritional status and geriatric diseases. The health education course consists of twelve 30-min lectures covering three topics. Every 2 weeks, healthcare professionals with a nutrition background deliver these lectures. Concurrently, social workers distribute brochures and booklets that condense the lecture notes into easily understandable tips and illustrations. Additionally, all lectures and printed materials are recorded and exhibited in the canteen, fostering the establishment of a healthy food environment.

**Table 1:** Elderly-targeted health education content

**Health education topics**	**Themes of courses**
Food and nutrients	Food categories and food diversity
	Energy and macronutrient needs for the elderly
	The role of micronutrients in healthy aging
	Good resources for protein
Chinese geriatric nutrition guideline	Malnutrition risk self-screening
	Dietary patterns for the elderly
	Food processing for seniors
	Dietary supplements
Nutritional status and geriatric-associated conditions	Hypertension
	Diabetes
	Osteoporosis
	Sarcopenia

#### Integrated nutrition support with personalized recommendations for the intervention group

Participants in the intervention group will receive integrated interventions that comprise three components: (1) health education with an emphasis on elderly nutrition, (2) personalized dietary advisory service powered by a knowledge-based meal recommendation system, and (3) a healthy food-providing service. Three roles are involved in this process: health professionals, community social workers, and community-dwelling elders. The community-dwelling elders serve as participants to improve their dietary behaviors. Meanwhile, the health professionals and social workers assist by facilitating the promotion of dietary changes through implementing health education and providing instructions on using the meal recommendation system.

#### Theoretical underpinnings

**Fig. 1:**
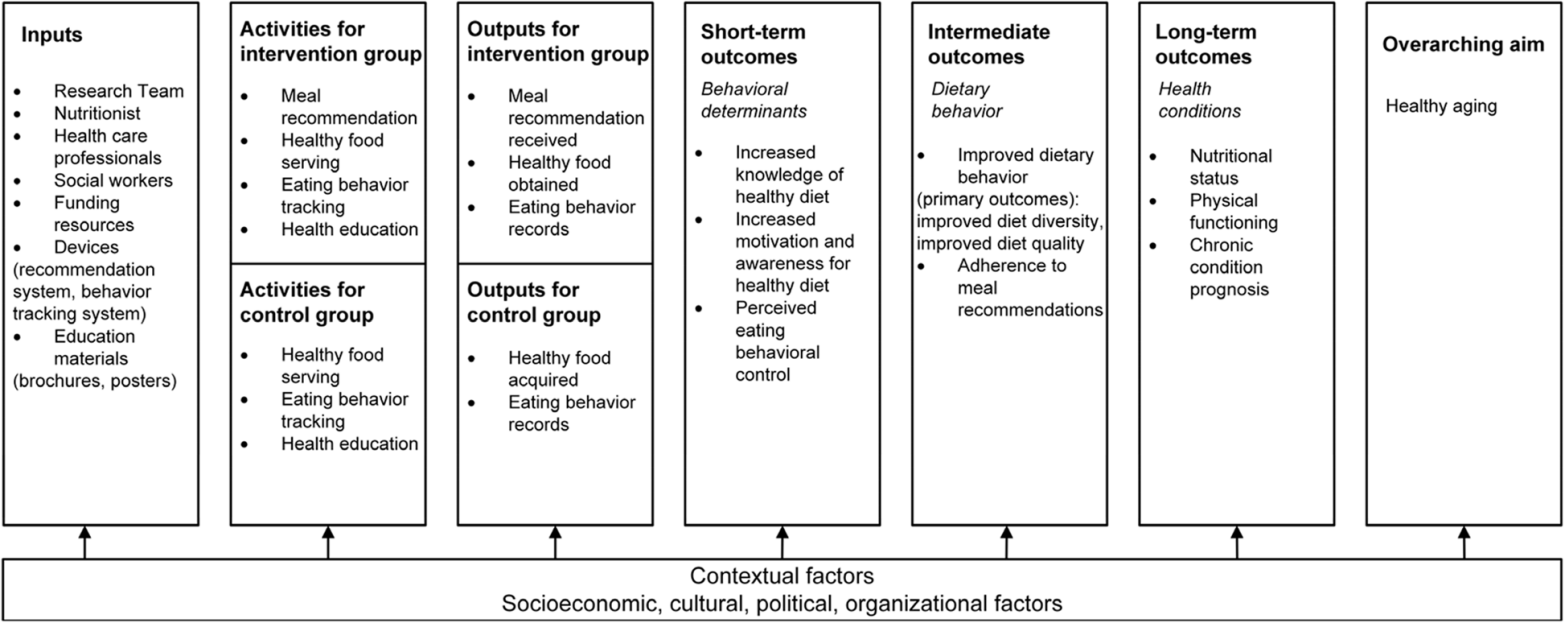
Logic model of dietary interventions targeting community-dwelling elders

A logic model is a visual roadmap that depicts an intervention study’s components, activities, and intended outcomes in the short term, medium term, and long term, meanwhile revealing the cause-and-effect linkage. Figure 1 shows the logic model of this study. The intervention components and outcome measures are included in the “activities” box. The expected short-term outcomes (knowledge, motivation, perceived behavioral control), medium-term outcomes (dietary behavior improvement, adherence to recommendations), and long-term outcomes (nutritional status, physical functioning, and chronic condition prognosis) were informed by the logic model.

#### Development and deployment of a meal recommendation system

**Fig. 2:**
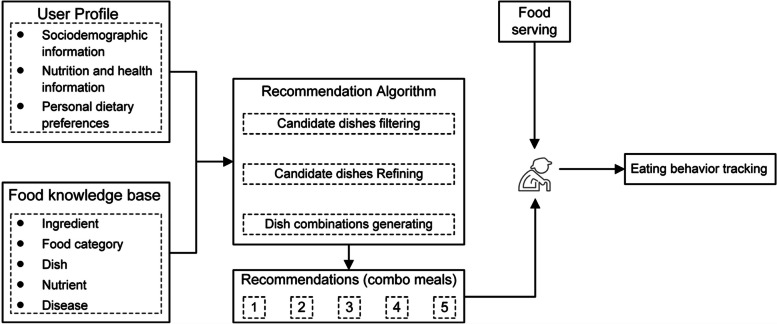
Personalized meal recommendation pipeline

The objective of a personalized meal recommendation system is to empower community-dwelling elders in their meal-planning endeavors by offering personalized food selection suggestions based on their dietary preferences and health conditions (Fig. 2). To incorporate the specific nutritional needs related to various medical conditions and individual dietary preferences, we have developed user-profiles and a food knowledge base to support the recommendation system.

User profiles are built to extract individualized dietary preferences and restrictions. Enrolled community-dwelling elders completed a baseline survey to collect their sociodemographic information, disease and health condition information, and dietary preferences. Additionally, the dietary behaviors of the community-dwelling elders will be tracked for 1 month under natural conditions before the formal recommendation procedure. This tracking period allows for the generation of their preferred dishes, which can be further transferred as another component of their personal dietary preferences. A food knowledge base has been developed and stored as a food knowledge graph, which encompasses information on five core concepts: dishes, ingredients, categories of ingredients, nutrients, and diseases, along with their corresponding attributes and intercorrelations. The knowledge base comprises a total of 180 dishes made from 120 ingredients, encompassing 27 essential nutrients. Furthermore, 30 common geriatric diseases were included to establish disease-related nutrient rules to build the system’s recommendation capabilities. The recommendation algorithm can be divided into three steps: filtering inappropriate dishes, refining preferred dishes, and generating candidate combo meals (a combination of several dishes) generation. First, diseases and health condition information are acquired from user profiles, so that disease-related constraining rules can be used to filter out the candidate dishes containing inappropriate ingredients for the elder individuals. Second, personal dietary preferences, present as top frequently selected dishes, are extracted from tracked eating behavior history to further help pop up most similar candidate dishes in terms of ingredients. Finally, several candidate dishes from different categories are combined as a meal (combo meal). Among all candidate combo meals, the top 5 combo meals will be selected as recommendation outputs according to the nutrient adequacy following the elderly nutrition guideline. The entire recommendation system will be deployed in the checkout system at a community canteen that serves community-dwelling elders on weekdays. During the intervention period, each elder will be given a card to swipe for identification at the beginning of the food selection process. At the same time as identification, they will receive a reminder informing them of five sets of recommended combo meals. Despite the provided dietary suggestions, participants will still be able to choose their preferred dishes voluntarily.

### Criteria for discontinuing or modifying allocated interventions {11b}

The assigned interventions in either group will be discontinued upon the subjects’ request to withdraw from the trial. No modification of the intervention is anticipated throughout the trial.

### Strategies to improve adherence to interventions {11c}

In this trial, adherence primarily refers to the participants’ acceptance and compliance with the recommendations. The degree of their adherence can be objectively measured by the concordance between the actual food choices of elderly individuals and the recommendations provided by the system. Several actions can be taken to enhance compliance, including (1) designing an elderly-friendly interaction process for food recommendations, (2) organizing successive health education courses to boost motivation and raise awareness among community-dwelling elders regarding the importance of maintaining a healthy diet, (3) providing regular updates on personal dietary behavior as feedback and as a trigger for further improvement, and (4) conducting follow-ups by healthcare professionals to enhance adherence to the interventions.

### Relevant concomitant care permitted or prohibited during the trial {11d}

No concomitant care or interventions are not allowed during the trial.

### Provisions for post-trial care {30}

Post-trial care is not applicable for this trial.

### Outcomes {12}

The primary outcome is the dietary behavior changes, specifically defined as dietary diversity, diet quality, and adherence to dietary interventions, from baseline (T0) to 3 months (T1) and 6 months (T2). The key secondary outcome is the perceived acceptability and usability of personalized meal recommendations as a dietary intervention specifically in the intervention group. The exploratory outcomes are nutritional status and anthropometric measurements of the community-dwelling elders.

### Participant timeline {13}

**Fig. 3:**
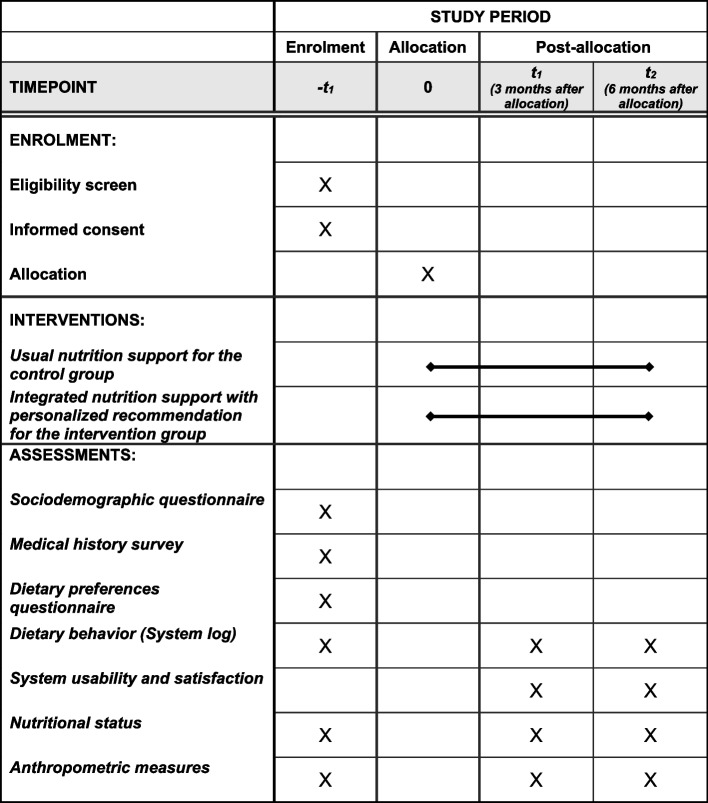
SPIRIT figure

**Fig. 4:**
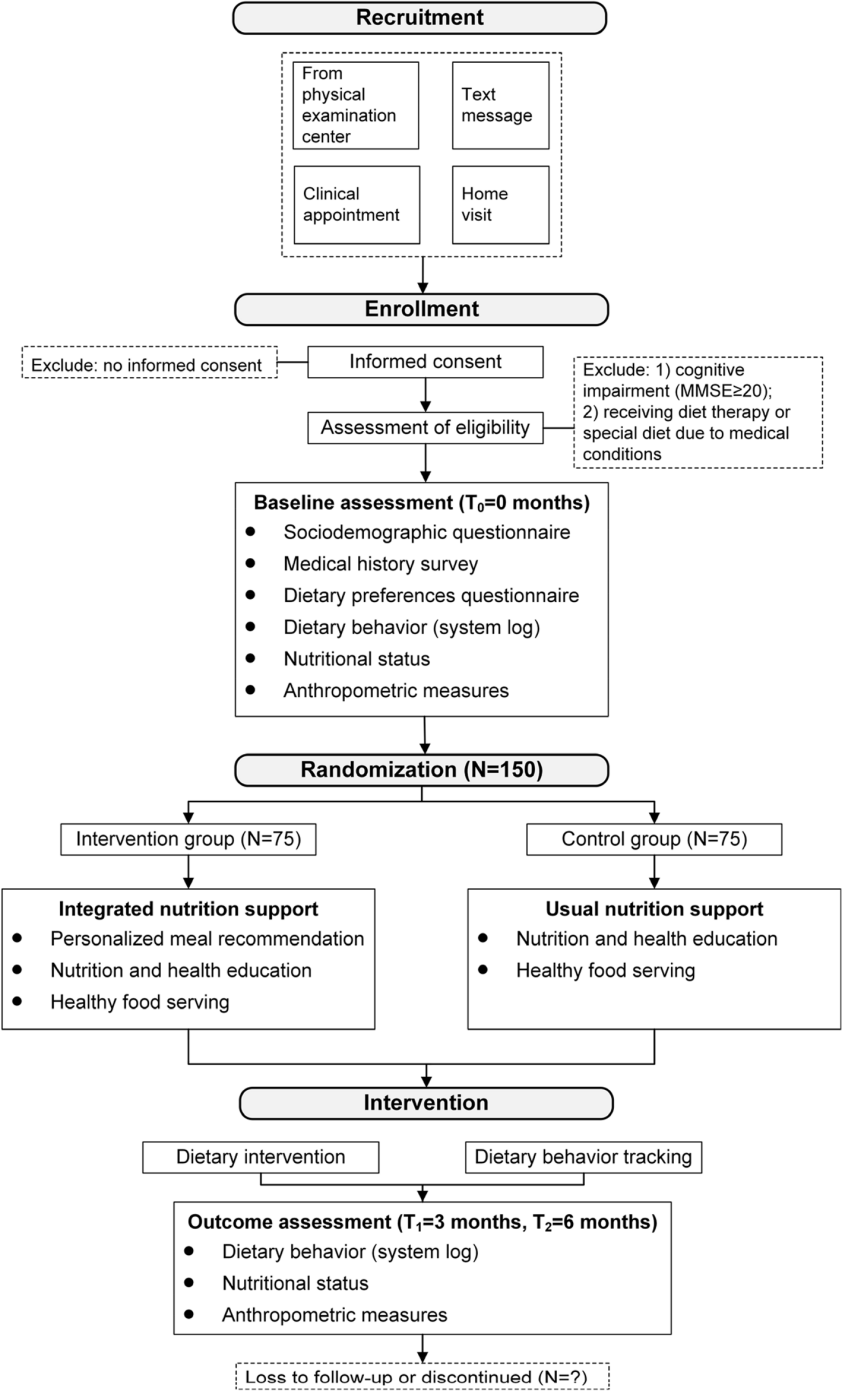
Timeline diagram

The SPIRIT figure is presented in Fig. 3, which provides an overview of the schedule of enrolment, interventions, and assessments. Figure 4 shows the timeline diagram.

### Sample size {14}

This study uses a randomized, non-blinded, two-armed study design with three times measurements during the study. The power calculation is based on the primary outcome measure- dietary diversity score. The sample size was calculated according to the repeated-measures formula [24, 25]: *N* = $\frac{2{({{\text{Z}}}_{1-\mathrm{\alpha }/2}+ {{\text{Z}}}_{1-\upbeta })}^{2} {\upsigma }^{2}}{k{\updelta }^{2}}$. In this formula, parameters denoting the number of repeated times *k* = 3, type I error rate *α* = 5% (two-sided), and a power level of 0.80. We set the smallest detected meaningful difference *δ* = 2.3 according to experts’ experience. Based on the results of relevant studies and our baseline survey, the correlation coefficient among measures *ρ* = 0.7, and equal standard deviation *σ* = 6.12 was used [24, 26]. It was estimated that the desired number of included participants at baseline should be set at a minimum of 60 for each arm. Consequently, a total of 150 community-dwelling elderly individuals are expected to recruit at baseline considering an attrition rate of 20% [27–29].

### Recruitment {15}

A convenience sampling method will be used to recruit community-dwelling elders from six communities mentioned in section {8}. Patients will be primarily recruited through three methods: (1) posting flyers at primary care centers during regular physical examinations of community-dwelling elderly individuals, (2) recommendations from community healthcare professionals during clinical appointments at community health service centers, (3) recommendations during community healthcare professionals’ regular home visits with elderly individuals. Additionally, the staff of the community health service center sent recruitment messages. We expect the physical examination to serve as the primary recruitment channel. Trained social workers will assist potentially eligible patients in confirming their eligibility. Eligible elderly individuals will receive invitations to attend the enrollment session, during which the social workers will provide instructions on participation procedures, and interested individuals will be given a consent package.

## Assignment of interventions: allocation

### Sequence generation {16a}

Under the cluster randomization design, one investigator will generate a randomized allocation sequence using R 4.2.0 to allocate the intervention and control sites at a 1:1 ratio. The investigator tasked with generating the randomization sequence is specifically chosen for non-involvement in the recruitment of centers or the collection of participant data.

### Concealment mechanism {16b}

Due to the utilization of a cluster randomization method, we do not conceal the allocation. As a result, the proposed concealment mechanism is not applicable in this scenario. The allocation sequence will be securely concealed and not disclosed until all centers are recruited and baseline data are collected.

### Implementation {16c}

An investigator will use a randomization algorithm to allocate six candidate sites to different groups, ensuring random and unbiased assignment. The results will be informed to the social workers at respective sites. At each site, social workers will be responsible for recruitment and conducting baseline assessments. All subjects who have provided informed consent will be included in the trial. The social workers will guide the enrollment session, explaining the intervention assignment process to the participants. Following enrollment, the overarching purpose of the study, intervention procedures in the assigned group, and measurement details will be thoroughly explained to each participant. Additionally, each participant will undergo a baseline assessment that includes collecting information on socio-demographics, dietary behaviors, anthropometric characteristics, medical history, and nutritional status.

## Assignment of interventions: blinding

### Who will be blinded {17a}

Due to the characteristics and design of interventional studies, participants and researchers were not blinded to interventions; data analysts, however, will be blinded to the assignment of interventions.

### Procedure for unblinding if needed {17b}

Blinding is not applicable to this trial.

## Data collection and management

### Plans for assessment and collection of outcomes {18a}

All primary and secondary outcomes will be repeatedly measured at baseline, 3 months, and 6 months. System usability and satisfaction will only be evaluated in the intervention group, while dietary behaviors (dietary diversity and diet quality, adherence to the interventions), nutritional status, and anthropometric measurements will be evaluated in both the intervention and control groups. Detailed information on data collection methods is shown in Table 2.

**Table 2:** The outcome measures and collecting methods

**Outcome measure**		**Collection method**
Dietary behavior	Dietary diversity	IoT system log, DDS
	Diet quality	IoT system log, CDGI-E
	Recommendation adherence	IoT system log
System usability and satisfaction	Satisfaction ratings	Questionnaire (CSAT)
	Ease of use ratings	Questionnaire (CES)
Nutritional status	Risk of malnutrition	Questionnaire (MNA-SF)
Anthropometric measurements	Body mass index	Basic body measurement (body weight scale, height rod)
	Waist circumference	Basic body measurement (measuring tape)
	Systolic blood pressure	Blood pressure monitor
	Diastolic blood pressure	Blood pressure monitor
	Skin folds	Basic body measurement (skinfold caliper)

### Dietary behavior

The assessment of dietary behavior encompasses three main aspects: dietary diversity, diet quality, and adherence to recommended meals. These three components are calculated based on the actual eating behaviors of elderly individuals. To track the food selections of community-dwelling elders, an IoT-based system is deployed in this trial. This system allows for the simultaneous tracking of the dishes chosen by elderly individuals for each meal, along with the check-out process. Dietary diversity will be evaluated using the dietary diversity score (DDS) [30], which has been successfully utilized to assess dietary diversity among the Chinese elderly population [31]. The diet quality of enrolled community-dwelling elders will be evaluated using China Elderly Dietary Guidelines Index (CDGI-E) [32], which is developed based on the elderly-related diet recommendations of the 2016 Chinese Dietary Guideline [33]. CDGI-E has been employed as a dietary quality evaluation index in a large-scale cross-sectional survey conducted nationwide to assess the dietary quality of the elderly [32]. Recording the actual eating behaviors allows investigators to determine the number and quantity of specific food categories in selected dishes. This information facilitates the calculation of the DDS and CDGI-E scores. Adherence to recommended meals is assessed by comparing the dishes selected by elderly individuals with the recommended ones, and the degree of adherence is recorded as the average number of matching choices to the total choices pre week.

### System usability and satisfaction

System usability and satisfaction are assessed using the ratings of satisfaction and ease of use from the intervention group. Modified Customer Satisfaction Score (CSAT) [34] on a 1 to 5 Likert scale (very unsatisfied, unsatisfied, neutral, satisfied, very satisfied) will be adopted to measure the degree of satisfaction among elderly individuals when using the recommendation system. At the end of each month, a question will be asked “How would you rate your overall satisfaction with the meal recommendation service you received?” to capture their overall satisfaction with the meal recommendation service. The perceived ease of use for the recommendation system among elderly individuals will be measured using a modified Customer Effort Score (CES) [35] on a 1 to 5 Likert scale (very difficult, difficult, neutral, easy, very easy). A question asking “On a scale of “very easy” to “very difficult,” how easy was it to understand the recommendations” will be posed at the end of each month.

### Nutritional status and anthropometric measurements

The nutritional status of community-dwelling elders will be assessed using the Mini Nutritional Assessment – Short Form (MNA-SF) [36], which offers a straightforward and efficient method for screening elderly individuals who may be malnourished or at risk of malnutrition. Anthropometric measurements will be obtained through basic body measurements and clinical blood pressure assessments. Table 2 provides a comprehensive list of the specific measuring equipment and devices used in this study.

### Plans to promote participant retention and complete follow-up {18b}

We will employ several retention strategies to minimize attrition. They include (1) confirming contact information at each assessment and obtaining contact information of two or more people who will know how to contact them; (2) providing a token of appreciation for each assessment, in addition to a small bonus gift for completing all assessments;(3) maintaining a carefully detailed tracking system with the information from both surveys and primary care center records to schedule follow-up assessments and to keep track of other retention efforts such as giving out small gifts; (4) providing daily necessities and health education materials helpful to healthy aging valued at around ¥100 (US $15) will be offered as compensation per assessment to encourage engagement and adherence of participants. In addition, we have conservatively estimated a 20% retention rate at the follow-up assessment for each study arm based on the high reported attrition rates in previous digital health interventions.

### Data management {19}

In this trial, assessment data will be collected through two methods: (1) an IoT-based ambient dietary behavior tracking system and (2) face-to-face evaluations. The assessment data will be electronically saved in an encrypted database and deidentified, with access granted only to researchers designated to control data quality and perform data analysis. Other investigators will only have read permission for the database. Double data entry and logic checks will be conducted to identify any suspicious submissions. Any suspected data errors or items with abnormal values will be logged and excluded from the final analysis. If necessary, investigators will make attempts to re-collect missing static data, such as sociodemographic and medical history. The entire process of data entry, recoding, auditing, and storage will be logged in documents, which are available from the corresponding author upon reasonable request.

### Confidentiality {27}

All records containing personal information will be stored separately in the database and will only be shared within the study for research purpose. Only healthcare professionals and investigators have access to the information necessary for conducting the intervention. Data analyst will be blinded to the allocation of groups.

### Plans for collection, laboratory evaluation, and storage of biological specimens for genetic or molecular analysis in this trial/future use {33}

There is no plan for biological specimen collection and analysis in this trial.

## Statistical methods

### Statistical methods for primary and secondary outcomes {20a}

Statistical analysis will be conducted using R (version 4.2.0; R Foundation for Statistical Computing). We will use a two-sided significance level of 5% and report the results with two-sided 95% confidence intervals (CIs) for all statistical analyses. For continuous variables, the Shapiro-Wilk test and Levene’s test will be utilized to identify the appropriate data treatment (i.e., parametric or non-parametric analysis), followed by a descriptive analysis presenting as means (SD) or median (IQR). Categorical variables will be presented as frequencies (percentages). For the statistical analysis of repeated outcome measures, generalized linear mixed models (GLMMs) will be used. These models are well-suited for our study design as they can effectively handle the complexities of repeated measures data and accommodate various response variable distributions, including normal, nonnormal, and ordinal categorical responses [37–39]. The primary fixed effects in our models will be the intervention and the time. To account for individual variability among participants, random effects will be included in the model to capture the individual variation. We will adjust for potential confounders that might influence the relationship between the intervention and the outcomes. These confounders include both basic demographics (age, gender, and education level) and heatlh-related factors (physical activity level, diet, smoking status, and medication use). We will further consider the inclusion of interaction terms in our model to examine if the effect of the intervention varies across different levels of confounders.

### Interim analyses {21b}

Formal interim analysis of the primary and secondary outcomes is not applicable in this trial.

### Methods for additional analyses {20b}

Subgroup analyses of the primary and secondary outcomes stratified by variables as follows: gender (male and female) and age group (< 75 years old, ≥ 75 years old). Adjusted GLMMs will be utilized in each subgroup to ensure that the effects observed in subgroups are adjusted for the same confounders as in the general analysis.

### Methods in analysis to handle protocol non-adherence and any statistical methods to handle missing data {20c}

Both intention-to-treat analysis (ITT) and per-protocol (PP) analysis will be conducted. Specifically, the interpretation of the intervention effect would be more likely to consider ITT as the gold standard approach. However, both ITT and PP analyses will be reported alongside each other for comparison. To conduct ITT analysis with incomplete data, missing data will be imputed using multiple imputation techniques.

### Plans to give access to the full protocol, participant-level data, and statistical code {31c}

The individual-level dataset and statistical code generated during the current study will be available from the corresponding author upon reasonable request after 5 years since the trial is finished.

## Oversight and monitoring

### Composition of the coordinating center and trial steering committee {5d}

A trial steering committee and a data monitoring committee have been established to oversee the trial. The steering committee consists of investigators, information scientists, and nutrition scientists from Medical Information Institution of the Chinese Academy of Medical Sciences, Chinese Center for Disease Control and Prevention, and Peking Union Medical College Hospital. The main responsibility of the steering committee is to evaluate the protocol to improve its feasibility and make necessary amendments to address ethical concerns. They also guide the planned interventions for the targeted elderly population.

### Composition of the data monitoring committee, its role and reporting structure {21a}

The data monitoring committee (DMC) comprises data scientists and statisticians from Peking Union Medical College. Their primary responsibility is to ensure the data quality of the study by conducting audits of the trial every 3 months. The DMC operates independently of the sponsor with no competing interests. The committee charter is subject to further review upon request from the corresponding author.

### Adverse event reporting and harms {22}

Adverse events in this trial include deaths, emergency room visits, onset of cognitive impairment, onset of physical dysfunction, and hospitalization lasting longer than 24 h. The investigators will collect and record the adverse events at the end of the study and then examine the association between their incidence and the health conditions of elderly individuals.

### Frequency and plans for auditing trial conduct {23}

The trial conduct will be audited by DMC every 3 months. The auditing process will be conducted independently of both the investigators and the sponsor.

### Plans for communicating important protocol amendments to relevant parties {25}

All protocol amendments will undergo review by the steering committee and IRB. Subsequently, the investigators, sponsors, healthcare professionals, social workers, and trial participants will be duly informed regarding the modifications.

### Dissemination plans {31a}

We will disseminate our study results via peer-reviewed journals, trial registers, and social media platforms like X/Twitter. A lay summary will be provided to participants for accessible understanding. If the trial yields successful outcomes, we plan to host patient and stakeholder workshops to further engage the community with our findings.

## Discussion

This research protocol describes the design of a personalized intervention aiming to improve the dietary behaviors of community-dwelling elders. In comparison to previous studies, our approach offers several distinct advantages. First, we utilize a personalized meal recommendation system tailored to the specific needs of community-dwelling elders. This recommendation system is driven by an advanced algorithm and a comprehensive nutrition knowledge base derived from guidelines, ensuring consideration of both disease-related nutrition requirements and dietary preferences. Secondly, our implementation plan uses a hybrid approach, incorporating health education and a food-serving program to maximize the potential benefits of the intervention. Third, we have implemented a user-centered design by providing personalized meal recommendations in the form of packaged meals. This approach reduces the cognitive burden on elderly users and enhances their adherence to the dietary recommendations.

The evaluation design is expected to comprehensively examine the effects of the intervention and its implementation process. A logic model has been established for the linkage between process indicators and anticipated outcomes. Our data collection strategy includes both objective and subjective measures, which will provide valuable insights into the effectiveness, acceptability, and usability of the proposed intervention. For example, objectively tracked eating behavior data are used to understand how well the elderly individuals adhere to the interventions, and their ratings will indicate their perception of the ease of use of the intervention delivery approach. Furthermore, our proposed pipeline of intervention implementation and evaluation is durable, whose extensions can be applied to improve other self-management behaviors in the elderly population, such as medication adherence, physical activities, and follow-up appointments. Exploration in terms of long-term effects and maintenance of our proposed intervention can be further examined based on our current study guided under RE-AIM framework [40].

The current research protocol has certain limitations. Elderly participants are likely to have a low acceptance of our innovative intervention, resulting in high attrition rates and low adherence to recommendations. Moreover, our intervention study design lacks blinding, and there will be extensive interaction between healthcare professionals and elderly participants, which may introduce the observer effect. To address these potential biases, we intend to mitigate them through comprehensive training for investigators and healthcare professionals. Additionally, we plan to establish significant bonus mechanisms to enhance participant adherence. Throughout the study period, the steering committee will provide supervision of intervention procedures to minimize protocol deviations. Lastly, our study’s primary focus is on dietary interventions, which overlooks the significant benefits of physical rehabilitation and bio-psycho-social approaches in geriatric care [41, 42]. Future research should thus adopt a more holistic approach to meet the complex health needs of the elderly population.

In conclusion, this study seeks to provide valuable insights into the effectiveness, acceptability, and usability of a personalized dietary intervention that utilizes data-driven insights for community-dwelling elders. Our results will inform the future development and integration of digital health strategies in primary care settings for the elderly, ultimately facilitating healthy aging.

## Trial status

This study protocol was approved on August 20, 2023, and this manuscript details the protocol in the original version. The first participant was enrolled on August 21, 2023, and recruitment is expected to be completed by October 19, 2023.

### Supplementary Information

**Additional file 1.**

## Data Availability

The trial datasets generated in this study are not publicly accessible to ensure the protection of individual privacy. Nevertheless, interested parties may obtain access to these resources by making a reasonable request to the corresponding author.

## Abbreviations

MCCs: Multiple chronic conditions
ADLs: Activities of daily living
MMSE: Mini mental state examination
DDS: Dietary diversity score
CDGI-E: China Elderly Dietary Guidelines Index
CSAT: Customer Satisfaction Score
CES: Customer Effort Score
MNA-SF: Mini Nutritional Assessment – Short Form
